# Maternal depression and child psychopathology among Attendees at a Child Neuropsychiatric Clinic in Abeokuta, Nigeria: a cross sectional study

**DOI:** 10.1186/s13034-016-0115-6

**Published:** 2016-09-02

**Authors:** Adeniran O. Okewole, Abiodun O. Adewuya, Ademola J. Ajuwon, Tolulope T. Bella-Awusah, Olayinka O. Omigbodun

**Affiliations:** 1 Child and Adolescent Unit, Neuropsychiatric Hospital, Aro Abeokuta, Nigeria; 2 Department of Behavioral Medicine, Lagos State University College of Medicine/Lagos State University Teaching Hospital, Ikeja, Lagos, Nigeria; 3 Department of Health Promotion and Education, University of Ibadan, Ibadan, Nigeria; 4 Centre for Child and Adolescent Mental Health, University of Ibadan, Ibadan, Nigeria

**Keywords:** Depression, Psychopathology, Maternal mental health, Child behavioural problems

## Abstract

**Background:**

Children with recognized, diagnosable mental and neurological disorders are in addition prone to emotional and behavioral problems which transcend their specific diagnostic labels. In accessing care, these children are almost invariably accompanied by caregivers (usually mothers) who may also have mental health problems, notably depression. The relationship between child and maternal psychopathology has however not been sufficiently researched especially in low and middle income countries.

**Methods:**

Mothers (n = 100) of children receiving care at the Child and Adolescent Clinic of a Neuropsychiatric Hospital in Abeokuta, Nigeria took part in the study. To each consenting mother was administered a sociodemographic questionnaire and the Patient Health Questionnaire, while information regarding their children (n = 100) was obtained using the Strengths and Difficulties Questionnaire. Data analysis was done with the Statistical Package for Social Sciences (SPSS) version 16.

**Results:**

The mean ages of the mothers and children were 40.4 years (SD 4.7) and 11.6 years (SD 4.1), respectively. Among the children, 63 % had a main diagnosis of seizure disorder. Regardless of main diagnosis, 40 % of all the children had a comorbid diagnosis. Among the mothers, 23 % had major depressive disorder. A quarter (25 %) of the children had abnormal total SDQ scores. A diagnosis of major depressive disorder in mothers was associated with poor total SDQ scores and poor scores in all SDQ domains except the emotional domain for the children. Major depressive disorder among the mothers was associated with not being married (p = 0.004; OR = 0.142, 95 % CI 0.037–0.546) and longer duration of the child’s illness (p = 0.039, OR = 1.165, 95 % CI 1.007–1.346).

**Conclusion:**

The study showed notable rates of depressive illness among mothers of children with neuropsychiatric disorders. Marked rates of emotional and behavioral disorders were also found among the children. Associations were found between maternal and child psychopathology. Mothers of children with neuropsychiatric disorders should be screened for depressive illness.

## Background

According to the World Health Organisation (WHO), maternal mental health is ‘‘a state of well-being in which a mother realizes her own abilities, can cope with the normal stresses of life, can work productively and fruitfully, and is able to make a contribution to her community’’[1]. Among the threats to maternal mental health are mood disorders, to which women are vulnerable at times of life cycle related hormonal challenge (e.g. the premenstruum, pregnancy, post-miscarriage, postpartum, and perimenopause). Neurobiological, genetic and psychosocial substrates underlie the increased vulnerability for depression in women [2].

In low and middle income countries (LMICs), studies suggest that rates of maternal depression are as high as 15–28 % in Africa and Asia (including 18.6 % in Nigeria), 50 % in Bangladesh, 28–57 % in Pakistan, and 35–47 % in Latin America [3, 4]. These figures largely represent perinatal depression. While perinatal depression is often the focus of attention, beyond the perinatal period represents a time when women remain at risk for a depressive disorder [5]. A particularly vulnerable group is mothers of children with chronic health problems. Such children require that their care be overseen by caregivers (usually mothers) who may also have mental health problems. A variety of studies have highlighted the psychological distress and morbidity associated with caring for children with mental disorders [6, 7]. Caregiving is associated with a range of psychological and emotional problems, as reported among Tanzanian mothers [8], including depressive symptoms, as reported among Latina mothers of children with developmental disabilities [9].

The impact of maternal depression on the physical health of the child has been well documented, especially in low and middle income countries [3, 10–13]. Studies conducted mainly among mothers with depression have also sought to demonstrate an association between maternal mental health and the mental health of the child [14–18]. A series of reports from the sequenced treatment alternatives to relieve depression (STAR*D) study provide a narrative of the negative impact of maternal depression on the psychological welfare of the child [19–22].

Mechanistic and methodological issues have however been raised—most notably the suitability of depressed mothers as informants on the emotional and behavioural status of their children—regarding these findings [23], and the contribution from Africa remains low. Studies addressing maternal and child mental health are rare in Africa due to shortage of researchers, heavy patient load, lack of funding, poor data collection and difficulty following up patients and their mothers. There is need for context-specific research to influence clinical practice and policy directions on the relationship between maternal and child mental health in LMICs. This study therefore aimed to investigate the relationship (if any) between maternal depression and child psychopathology among attendees at a specialist child and adolescent mental health facility in Nigeria.

## Methods

### Study location

The study was conducted at the Child and Adolescent Clinic (CAC) of the Neuropsychiatric Hospital, Aro, Abeokuta, Ogun State, Nigeria. The Child and Adolescent Clinic became functional in 2007 and is run by the Child and Adolescent Unit of the Hospital which is managed by three consultant psychiatrists. Resident doctors rotate through the unit, with a locum consultant neurologist seeing patients at the clinic once a week. There is a full complement of twenty multidisciplinary staff providing care in the clinic including doctors, nurses, occupational therapists, speech and language therapists, and pharmacists, with access to social workers, psychologists and physiotherapists. Clinics are run twice a week, with an average of 25 children seen at each clinic. A brief review of the records showed that 90 % of carers are Mothers, and as much as 60 % of children seen have epilepsy, either occurring alone or comorbidly with another disorder. Other commonly seen disorders include intellectual disability, autism spectrum disorders, attention deficit hyperactivity disorder, mood disorders, and early onset psychosis.

### Study population and sampling

The study population comprised mothers of children receiving treatment at the CAC. Included mothers were those whose children had illness of longer than 6 months’ duration, and who were the primary caregivers (meaning those who were living with the child receiving treatment, were financially responsible for the care of the child, and were called upon in emergencies involving the child). Mothers with prior lifetime history of mental illness (who had been diagnosed with mental illness at any time before the study, either before or after the child was born), or who reported having a family history of mental illness, were excluded. This was done given that a number of mothers may have suffered depression even without having a child with a mental or neurological illness, and the study design tried to exclude such to better address the question of a relationship between maternal depression and child psychopathology.

The study participants were recruited using a systematic random technique. On every clinic day, a random start was picked by a simple ballot from the first two children presenting at the clinic. Thereafter, alternate children accompanied by the Mother were picked. Those who were not accompanied by their Mothers, or for whom consent was not obtained, were replaced by the next suitable mother. This process gave ten mothers to be interviewed per clinic day, or twenty per week, over a period of 5 weeks in March through April, 2015.

### Study instruments and administration

Three instruments were used to collect data. These were:

1. A questionnaire containing socio-demographic details of the mother and child, as well as relevant clinical details of the child such as diagnoses and duration of illness.
2. Patient Health Questionnaire, PHQ-9 (all mothers): this was used to make diagnosis of depression among the mothers. It is a nine-item self-administered questionnaire by Kroenke et al. [24] The PHQ-9 has been validated for use in Nigerian populations for screening for minor and major depressive disorder by Adewuya et al. [25] who reported that the PHQ-9 had good internal consistency of 0.85 and good concurrent validity with the Beck’s Depression Inventory (r = 0.67, p < 0.001). Using the receiver operating characteristic (ROC) curve, the authors reported that the optimal cut-off score for minor depressive disorder is 5 (sensitivity 0.897, specificity 0.989, positive predictive value—PPV 0.875, negative predictive value—NPV 0.981 and overall correct classification—OCC rate 0.973) while for major depressive disorder only is 10 (sensitivity 0.846, specificity 0.994, PPV 0.750, NPV 0.996 and OCC rate 0.992).
3. Strengths and Difficulties Questionnaire, SDQ (all children): The SDQ is a brief screening tool by Goodman et al. [26] for behavioral problems in children and adolescents. SDQ contains twenty-five item questions and five clinical sub-scales: emotional symptoms, conduct problems, hyperactivity, peer problems and pro-social behavior. The SDQ has been previously used in Nigeria by Bakare et al. [27].

The PHQ and the SDQ are available in Yoruba, the language widely spoken in the study area. The Yoruba versions were required because of the assumption that not all subjects would be fluent in English. Participants were recruited from among mothers of children presenting at the CAC. On the designated clinic days, Mothers to be recruited into the study were picked from the pool presenting on each clinic day. They were approached on the morning of the clinic while waiting for their children to be seen. Those who provided consent were recruited. All mothers were given the socio-demographic questionnaire, PHQ-9 and SDQ to fill while awaiting consultation. Mothers who were unable to read or write had the questionnaire read to them by the investigator.

### Ethical considerations

Ethical approval for the study was obtained from the Health Research Ethics Committee of the Neuropsychiatric Hospital, Aro Abeokuta. All mothers signed written consent forms after the nature, purpose and scope of the study had been explained to them. Verbal assent was also obtained from the children, who were physically present when their mothers were being interviewed. Although the children were not interviewed directly, their mothers were required to supply information about them. No age limit was adopted for this.

### Data management

A spreadsheet was used for initial data recording from the various instruments. The prevalence of depression and socio-demographic variables was presented using descriptive statistical measures such as means (with standard deviations) and frequency tables. On the PHQ, a score of 5 and above (out of a total of 27) was considered as screen positive for any depression, while a cut-off score of 10 and above was adopted as screen positive for major depressive disorder (MDD) only. This followed the cut-off points reported by Adewuya et al. [25] for minor and major depressive disorders respectively. The relationship between maternal depression and child emotional/behavioral problems was tested using Chi squares, t tests and correlations as appropriate. Scores for emotional/behavioral problems among the children, assessed by the SDQ, were computed as total scores and subscale scores for emotional, conduct problems, hyperactivity, peer problems and prosocial subscales [26]. The 25 items in the SDQ are divided into these 5 subscales with 5 items each. Items in each subscale are scored (0–10) after which the scores are categorized as normal, borderline or abnormal. A total score (0–40) is also generated from four out of the five subscales (excluding the prosocial subscale). However, inferential analysis for SDQ scores was done using raw scores (quantitative variables). For variables significantly associated with screening positive for major depressive disorder, logistic regression analysis was done. Similarly, linear regression was done for variables associated with scores on the SDQ. Tests were two-tailed, with level of significance set at p < 0.05. Statistical analysis was done using version 16 of SPSS.

## Results

### Sociodemographic and PHQ profile of the Mothers

In all, 100 mothers meeting criteria for inclusion participated in the study. The mean age of the mothers was 40.4 years (SD 6.14), ranging from 27 to 55 years. Other socio-demographic characteristics of the mothers are presented in Table 1. Majority of the mothers were married (85 %), Yoruba (91 %) and employed (95 %). Among the mothers, 41 % screened positive for depressive symptoms, while 23 % met the cut-off for a major depressive disorder.

**Table 1:** Socio-demographic profile of the mothers

Variable	Frequency	%
*Age (years)*		
26–35	28	28
36–45	55	55
46–55	17	17
*Marital status*		
Single	3	3
Married	85	85
Divorced/separated	7	7
Widowed	5	5
*Ethnicity*		
Yoruba	91	91
Ibo	5	5
Others	4	4
*Religion*		
Christianity	65	65
Islam	35	35
*Highest education*		
No formal	6	6
Primary	35	35
Secondary	43	43
Tertiary	16	16
*Employment status*		
Employed	95	95
Unemployed	5	5

### Sociodemographic and clinical profile of the children

The mean age of the children was 11.6 years (SD 4.1), and ranged from 4 to 17 years. The median duration of illness for the children was 5 years (interquartile range 7 years), while median duration of treatment was 1 year (interquartile range 1.5 years). The age and gender distribution, educational status and diagnoses of the children are presented in Table 2. Among the children, there was a male predominance. More than 60 % had a main diagnosis of seizure disorder, while 40 % had a comorbid disorder in addition to the main diagnosis.

**Table 2:** Sociodemographic and clinical profile of the children

Variable	Frequency	%
*Age (years)*		
0–9	77	77
10–17	23	23
*Gender*		
Male	53	53
Female	47	47
*Level of education*		
No formal	11	11
Nursery/primary	53	53
Secondary	34	34
Vocational/special	2	2
*Main diagnosis*		
Seizure disorder	63	63
Intellectual disability	26	26
Autism	3	3
ADHD	2	2
Psychosis	6	6
*Comorbid diagnosis*		
Seizure disorder	17	17
Intellectual disability	13	13
ADHD	2	2
Psychosis	6	6
Conduct disorder	1	1
Specific developmental disorder	1	1
No comorbid diagnosis	60	60

The mean total SDQ score of the children was 13.1 (SD 7.1), while mean scores in the different domains were as follows: emotional (2.5, SD 1.6), conduct (2.8, SD 2.3), hyperactivity (5.3, SD 3.2), peer problems (2.4, SD 2.3) and prosocial behavior (5.5, SD 2.5). Overall, a quarter (25 %) of the children had scores in the abnormal range. Over half were rated abnormal in the prosocial subscale, while abnormal scores in the hyperactivity and conduct problems subscales were found in 38 and 21 % respectively. However, only 5 and 1 % of the children respectively were rated abnormal on the peer problems and emotional subscales. The proportions of children with borderline scores were as follows: emotional (3 %), hyperactivity (6 %), conduct (12 %), peer problems (10 %), prosocial behavior (28 %), and total scores (10 %). Finally, the proportions of children with normal scores were as follows: emotional (96 %), hyperactivity (56 %), conduct (67 %), peer problems (85 %), prosocial behavior (18 %) and total scores (65 %).

### Relationship between maternal depressive illness and other Mother and child variables

Associations between screening positive for major depressive disorder and various maternal variables are shown in Table 3. A significantly larger proportion of non-married mothers were found to screen positive for major depressive disorder.

**Table 3:** Relationship between mothers’ diagnosis of major depressive disorder (MDD) and selected maternal variables

Variable	MDD	No MDD	Difference
*Age of Mother (years)*			
26–35	4 (14.3 %)	24 (85.7 %)	χ^2^ = 4.415, df = 2
36–45	12 (21.8 %)	43 (78.2 %)	p = 0.110
45–55	7 (41.2 %)	10 (58.8 %)	
*Marital status of Mother*			
Married	16 (18.8 %)	69 (81.2 %)	χ^2^ = 5.581, df = 1
Not married	7 (46.7 %)	8 (53.3 %)	p = 0.040*
*Maternal education*			
Primary/less	9 (22 %)	32 (78 %)	χ^2^ = 0.744, df = 2
Secondary	9 (20.9 %)	34 (79.1 %)	p = 0.730
Tertiary	5 (31.2 %)	11 (68.8 %)	

* p < 0.05

As shown in Table 4, children of mothers with major depressive disorder had significantly longer duration of illness. Mothers of children with seizure disorder were significantly less likely to be depressed compared to mothers of children with intellectual disability or other disorders.

**Table 4:** Relationship between mothers’ diagnosis of major depressive disorder (MDD) and selected child variables

Variable	MDD	No MDD n = 23 (%)	Difference n = 77( %)
*Age of child (years)*			
0–9	18 (23.4 %)	59 (76.6 %)	χ^2^ = 0.027, df = 1
10–17	5 (21.7 %)	18 (78.3 %)	p = 0.558
Duration of illness, Mean (SD)^a^	7.9 (4.9)	5.5 (4.6)	t = −2.09, p = 0.039*
*Child education*			
No/special	4 (30.8 %)	9 (69.2 %)	χ^2^ = 3.708, df = 2
Nursery/primary	15 (28.3 %)	38 (71.7 %)	p = 0.160
Secondary/more	4 (11.8 %)	7 (63.6 %)	
*Main diagnosis*			
Seizure disorder	9 (14.3 %)	54 (85.7 %)	χ^2^ = 7.32, df = 2
Intellectual disability	10 (38.5 %)	16 (61.5 %)	p = 0.020*
Other	4 (36.4 %)	7 (63.6 %)	
*Comorbidity*			
Present	11 (27.5 %)	29 (72.5 %)	χ^2^ = 0.762, df = 1
Absent	12 (20 %)	48 (80 %)	p = 0.469

* p < 0.05

^a^Duration in years

Table 5 shows associations between maternal depressive illness and the different domains of the SDQ. Significant differences were found between mothers with MDD and those without in their scoring of their children in all except the emotional domain. To check the possible effect of confounding, linear regression analysis was done with SDQ total and subscale scores separately as dependent variables, with maternal depression, duration of illness and main diagnosis enterredas covariates. As shown in Table 6, there remained a significant relationship between maternal depression and only the conduct subscale and total SDQ scores.

**Table 5:** Relationship between maternal major depressive disorder and child’s emotional/behavioural problems as assessed by the SDQ

SDQ domain	MDD (n = 23)	No MDD	t	Sig	95 % CI	
	Mean (SD)	Mean (SD)			Lower	Upper
Emotional	3.0 (1.2)	2.4 (1.7)	1.53	0.128	−0.17	1.30
Conduct	3.9 (2.4)	2.5 (2.1)	2.65	0.010*	0.34	2.41
Hyperactivity	7.1 (4.8)	3.4 (3.0)	3.10	0.003*^+^	0.81	3.70
Peer problems	3.5 (2.1)	2.7 (2.2)	2.64	0.010*	0.35	2.51
Prosocial behavior	4.4 (2.9)	5.8 (2.3)	−2.51	0.014*	−2.58	−0.30
Total scores	17.4 (6.3)	11.8 (6.8)	3.53	0.001*^+^	2.46	8.80

* p < 0.05

^+^p < 0.01

**Table 6:** Linear regression for children’s scores on SDQ

Variable	Beta	p value	95 % CI	
			Upper	Lower
*SDQ total*				
Main diagnosis	0.430	<0.001*^+^	2.648	6.210
Duration of illness	0.170	0.051	−0.006	4.819
Maternal MDD	0.201	0.025*	0.425	6.315
*SDQ emotional*				
Maternal MDD	0.085	0.411	−0.442	1.071
*SDQ hyperactivity*				
Maternal MDD	0.186	0.051	−0.005	2.822
*SDQ conduct*				
Maternal MDD	0.219	0.035*	0.086	2.251
*SDQ Peer problems*				
Maternal MDD	0.086	0.281	−0.399	1.357
*SDQ prosocial behaviour*				
Maternal MDD	−0.149	0.116	−1.963	0.219

Predictors: (Constant), main diagnosis of child, child’s duration of illness, and maternal major depressive disorder (MDD)

* p < 0.05

^+^p < 0.01

### Regression models for maternal depression

A significant association was found between maternal depression and the mothers’ marital status, the main diagnosis of the child, and the duration of illness of the child. To check the effect of confounding, these were entered separately into logistic regression with mother’s age, employment status, ethnicity, as well as the child’s age and gender as covariates. As shown in Table 7, a significant difference remained with marital status and duration of illness in the child (maternal age and age of the child contributed significantly to the two models respectively), but not with main diagnosis of the child.

**Table 7:** Logistic regression for major depressive disorder

Variable	Wald	p value	OR (95 % CI)
*Marital status*			
Not married	8.079	0.004*^+^	0.142 (0.037–0.546)
Married			Ref
Maternal Age	4.990	0.025*	1.136 (1.016–1.271)
*Main diagnosis*			
Epilepsy	1.871	0.171	0.162 (0.012–2.202)
Others			Ref
*Duration of illness*	4.248	0.039*	1.165 (1.007–1.346)
Age of child	5.718	0.017*	0.795 (0.659–0.960)

Covariates: maternal age, employment status, ethnicity, age of child, sex of child

* p < 0.05

^+^p < 0.01

## Discussion

The study examined the relationship between depressive illness among mothers of children with neuropsychiatric disorders, and the presence of emotional and behavioural problems among the children. Scores for depression among mothers was found to be associated with several domains of emotional and behavioural problems among the children.

With respect to the main diagnoses of the children, over 60 % of the children had seizure disorder. This is a finding which was reported at a similar facility in Lagos, Nigeria [28]. While it could be argued that childhood epilepsy should be treated by paediatric neurologists, this subspecialty is thin on the ground, with only one paediatric neurology facility in Abeokuta and two in Lagos. The pathway to care of most of the children presenting at the child and adolescent unit of a specialist psychiatric facility often takes them through traditional and spiritual healers rather than orthodox care centres [28]. The preference for a dedicated facility, rather than the pediatric neurology units which are embedded in paediatric services within a busy general medical facility, is also given as a recurrent reason by mothers for this preference. In addition, many children present with neuropsychiatric disorders with epilepsy as a comorbidity. In this study, nearly a fifth of children presented with epilepsy as a comorbid disorder. This agrees with prior reports that epilepsy, together with mental retardation, is a major presentation to child and adolescent mental health services in resource poor countries [28]. The generally high level of maternal education, which has also been reported to increase access to child and adolescent services [28], may be an additional factor in encouraging mothers not only of children with epilepsy but other disorders as well to access care.

Using the PHQ, 41 % of mothers screened positive for any depression, while 23 % screened positive for a major depressive disorder. These figures are higher than the 18.6 % prevalence of depression among a community sample of Nigerian mothers [4], which may reflect the fact that the PHQ used in this study is a screening tool, which may have captured a number of false positives. The proportion of depressed mothers in this study was however lower than the finding of about 50 % depression among caregiving mothers of children with mental health problems in the United States and Nigeria [29, 30]. The difference in prevalence may be explained by the fact that these other studies examined lifetime prevalence rather than current prevalence as assessed in this study. The implication of this finding is that a considerable proportion of mothers of children with neuropsychiatric disorders have to cope with depression in addition to caring for their children.

Maternal depression was found in this study to be associated with a longer mean duration of child’s illness. This finding agrees with the report by Rimehaug et al. [7] that emotional distress in mothers was associated with increased duration of the child’s illness. It is conceivable that having to cope with a challenging neuropsychiatric illness in a child wears down the mother’s defences and exerts an emotional toll.

While bearing in mind the small sample size of the non-married group, mothers who were not currently married (single, separated, divorced or widowed) were found to be more likely to be depressed. According to Laxman et al. [31], the presence of a literate father and responsive caregiving were associated with lower levels of depressive symptoms for mothers of children with an autism spectrum disorder. These resources are however not available to non-married mothers. The finding may also be linked, as previously postulated, to the lack of a confiding relationship which could be a risk factor for depression [32].

A higher proportion of major depressive disorder (more than a third) was also found among mothers of children with intellectual disability, while less than 15 % of mothers of children with seizure disorder had a major depressive disorder. Regression modeling revealed the likelihood of confounding in this association. Nevertheless, the finding may reflect the more severe and persistent symptomatology and the heavier demands that are associated with intellectual disability. However, several other studies have shown that the prevalence of depressive illness among mothers of children with epilepsy can be higher than for mothers in general [30, 33, 34].

Maternal depression scores on the PHQ were found to correlate positively with total SDQ scores, as well as with scores in all domains except emotional problems. Following regression analysis, this finding remained significant only for total SDQ scores and the conduct subscale. The findings of our study concur with those among Australian children with pervasive developmental disorders and developmental delay [6] and Australian children with intellectual disability [16] that child emotional and behavioural problems were associated with high rates of maternal mental health problems.

Rimehaug et al. [7] observed that maternal and child mental health problems were bidirectional, with maternal emotional distress being reported to increase with child externalising symptoms, while Boyd et al. [15] found that 25.4 % of children of mothers with depressive illness had clinical-range externalising symptoms. These agree with the finding of child conduct problem scores being correlated with scores for maternal depression in this study. The lack of relationship observed with respect to internalising emotional problems may reflect a generally low report of emotional problems among mothers of their children, rather than a true absence of association.

These findings suggest that children with neuropsychiatric disorders, who in addition have problems with social interaction, may constitute a source of distress for mothers who may then be vulnerable to depressive symptoms. On the whole, externalizing symptoms (particularly conduct problems) may be key features which characterize children with neuropsychiatric disorders whose mothers go on to develop depressive illness.

Following from this study, the authors wish to recommend that mothers of children with neuropsychiatric disorders should be routinely screened for depressive illness. An integrative approach leveraging maternal mental health care on platforms for caring for children with neuropsychiatric disorders should be adopted. A less restrictive and specialized approach to care, which includes management of neurodevelopmental disorders as well as neurological conditions such as epilepsy in the same model of care, may prove of universal benefit and not only in resource poor settings. It is noteworthy that community-based interventions such as the mental health gap action programme (mhGAP) have incorporated epilepsy as a priority condition alondside other mental illnesses. Such integration may also be desirable at the tertiary level.

This study provides information linking psychopathology among mothers and their children with neuropsychiatric disorders. The study was however limited by a cross-sectional design, which makes it difficult to determine the direction of causality. The question of whether maternal depressive illness precedes child psychopathology, or vice versa, or indeed whether the relationship is bidirectional, will require a longitudinal study design. Secondly, while the study was adequately powered, a study with a larger sample size would enable exploration of more variables in further statistical detail. Thirdly, while it was inevitable that a parental assessment be used especially for children with severe disabilities who were unable to volunteer information, the methodological implications of asking depressed mothers to provide information about their children has been pointed out [23]. The obvious solution may be to utilize clinician-administered tools for rating of depression and child psychopathology, rather than relying on the mothers’ reports. Future studies for instance of children with epilepsy may also utilise self-report questionnaires (to be filled by the children themselves) to obtain information especially about internalising symptoms. Although efforts were made to exclude mothers with a prior lifetime history of mental illness, this still does not entirely rule out the possibility of temporal overlap because the precise onset of depressive symptoms in relation to the onset of child symptoms and diagnosis could not be ascertained. Finally, the PHQ, though having excellent psychometric properties, is not diagnostic for depression. Other studies may choose to rely on a definitive diagnosis.

## Conclusions

The study reported notable rates of depressive illness among mothers of children with neuropsychiatric disorders. Factors associated with maternal depressive illness included mother’s marital status and longer duration of child’s illness. Marked rates of emotional and behavioural disorders were also found among the children, with children with longer duration of illness and children with a diagnosis of intellectual disability having more psychopathology. Associations were found between maternal and child psychopathology. It is therefore recommended that mothers of children with neuropsychiatric disorders should be routinely screened for depressive illness. An integrative approach leveraging maternal mental health care on platforms for caring for children with neuropsychiatric disorders should be adopted. Further studies involving maternal and child interventions and integrated systems of care are also required.

## Abbreviations

CAC: Child and Adolescent Clinic
LMIC: low and middle income countries
MhGAP: Mental Health Gap Action Plan
MDD: major depressive disorder
NPV: negative predictive value
OCC: overall correct classification
PHQ: patient health questionnaire
PPV: positive predictive value
ROC: receiver operating characteristics
SD: standard deviation
SDQ: strengths and difficulties questionnaire
SPSS: statistical package for social sciences
STAR*D: sequenced treatment alternatives to relieve depression
WHO: World Health Organisation
